# *Vibrio cholerae* and *Vibrio paracholerae* bacteraemia strains encompass lineages that share resistome and virulome profiles

**DOI:** 10.1590/0074-02760240159

**Published:** 2025-02-03

**Authors:** Sergio Mascarenhas Morgado, Erica Lourenço da Fonseca, Ana Carolina Paulo Vicente

**Affiliations:** 1 Fundação Oswaldo Cruz-Fiocruz, Instituto Oswaldo Cruz, Rio de Janeiro, RJ, Brasil

**Keywords:** blood, NOVC, septicaemia, third-generation cephalosporin, human infection

## Abstract

**BACKGROUND:**

Non-O1 and non-O139 *Vibrio cholerae* (NOVC) that cause bacteraemia have attracted the attention of the public health community around the world, mainly due to the prospect of outbreaks and the way to treat such infections.

**OBJECTIVES:**

To identify *V. cholerae* lineages and their antibiotic resistance and virulence factors associated with bacteraemia.

**METHODS:**

*Vibrio cholerae* genomes associated with strains isolated from blood were retrieved and subjected to core genome-based phylogenomic analysis with Roary. The virulome and resistome were searched with abricate using the VFDB and CARD databases.

**FINDINGS:**

Analysis showed that, in addition to *V. cholerae*, *Vibrio paracholerae* also causes bacteraemia. The NOVC group was highly diverse, although genomes from different countries were related. Most bacteraemic Vibrios came from countries not affected by epidemic/endemic cholera. The NOVCs virulome presented factors, such as type III and VI secretion systems, HapA, HlyA, RTX, and TLH. Importantly, no resistance to third-generation cephalosporin has been identified in the resistome of NOVCs.

**MAIN CONCLUSIONS:**

The presence of multiple NOVC lineages that cause bacteraemia in different parts of the world shows that there is no geographic and socioeconomic restriction for these cases. Therefore, healthcare systems need to be aware of this uncommon but deadly Vibrio infection.

*Vibrio cholerae* is a non-invasive bacterium generally recognised as the etiological agent of cholera diarrhoea, but eventually, this species also causes other clinical manifestations, such as gastroenteritis, skin and soft tissue, and other extraintestinal infections.^1^ In most of these cases, non-O1 and non-O139 *V. cholerae* (NOVC) strains are involved. More recently, reports of NOVCs causing bacteraemia have drawn the attention of the public health community around the world, mainly due to the prospect of outbreaks and the way to treat such infections.^1^ Regarding the treatment of NOVC infections, most cases have been successful with the use of quinolones, and also with dual-agent therapy, combining a third-generation cephalosporin with a tetracycline agent.^2^ ^,^ ^3^

NOVCs generally lack cholera toxin (CTX) and the toxin co-regulated pilus (TCP), however, some strains harbour other virulence factors that contribute to their pathogenicity and increase the risk of invasive infections, such as heat-stable enterotoxins, hemagglutinin proteases, haemolysins, and type III and VI secretion systems.^4^ These virulence factors may occur in some strains that cause invasive human infections, including bacteraemia, but also in other Vibrios strains.^4^

Therefore, as most reports of NOVC bacteraemia come from case studies,^5^ ^,^ ^6^ ^,^ ^7^ ^,^ ^8^ here we sought to analyse all available genomes of bacteraemia-associated NOVCs to identify possible lineages and the virulence determinants that would explain the invasive capacity of these NOVCs.

## MATERIALS AND METHODS

All *V. cholerae* genomes from Genbank associated with strains isolated from blood (n = 26) were retrieved (May 2024), and virulence and resistance genes were searched with Abricate (http://github.com/tseemann/abricate) considering identity and coverage of ≥ 60% using the VFDB and CARD databases, respectively.

The 26 genomes, in addition to other *V. cholerae* O1 genomes, were subjected to a core genome-based phylogenomic analysis using Roary v3.13.0 (https://github.com/sanger-pathogens/Roary), snp-dist v2.5.1 (https://github.com/sanger-pathogens/snp-sites), and IQtree v1.6.12 (https://github.com/Cibiv/IQ-TREE). The tree was drawn using iTOL webserver (https://itol.embl.de/). Genomes of *Vibrio paracholerae* were used as outgroups.

## RESULTS AND DISCUSSION

**Fig. 1:**
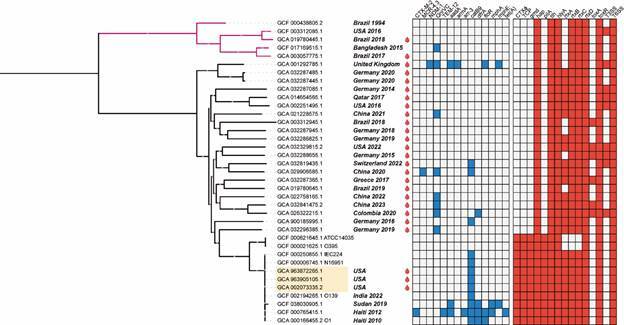
phylogenetic analysis of *Vibrio* genomes. Maximum-likelihood tree with 1000 ultrafast bootstrap replicates built based on alignments of *Vibrio cholerae* (black branches) and *Vibrio paracholerae* (pink branches) core genome. The best evolutionary model was GTR+F+ASC+R3, selected by the Bayesian information criterion. When available, each genome was associated with its location and year of isolation. The genomes of bacteraemia cases show an image of a drop of blood. Coloured blocks in the heatmaps indicate the presence of antibiotic resistance (blue) and virulence (red) genes. Red circles on branches represent > 70% bootstrap.

To contribute to the understanding of the increasing number of cases worldwide related to *V. cholerae* bacteraemia, we searched the Genbank database, revealing that 26/10,117 *V. cholerae* genomes could be considered cases of bacteraemia since *V. cholerae* were isolated from human/blood. However, the phylogenomic analysis showed that 2/26 were in fact *V. paracholera*e (GCA_019780445.1 and GCA_003057775.1) (Fig. 1, pink branches). *V. paracholerae* was recently described as a sister species of *V. cholerae*,^9^ also sharing a repertoire of virulence genes with NOVCs.^9^ ^,^ ^10^ Therefore, it is revealed that *V. paracholerae* is another Vibrio species to be considered in cases of bacteraemia, as well as *Vibrio vulnificus*, NOVC, and others.^8^

The phylogeny also showed that the majority of *V. cholerae* bacteraemia genomes were NOVC (n = 21), while three US genomes were serotype O1 (Fig. 1). In fact, NOVCs are known to cause extraintestinal infections, including bacteraemia.^1^ Importantly, these NOVCs did not represent lineages, indicating that a diversity of NOVCs would be associated with bacteraemia rather than specialised lineages. The exception was bacteraemia associated with *V. cholerae* O1, which occurred in the USA (n = 3) involving the 7th Pandemic strains (Fig. 1, beige background). The majority of genomes were from Europe (Germany, n = 8; UK, n = 1; Greece, n = 1; Switzerland, n = 1), USA (n = 5), and China (n = 4). Few others were from bacteraemia in Qatar (n = 1), Brazil (n = 2), and Colombia (n = 1) (Fig. 1). Interestingly, most of these countries are not epidemic/endemic to cholera. This limited number of countries reporting genomic data on *V. cholerae* bacteraemia strains is likely due to the lack of genomic investigation of these cases, particularly in low-income endemic/epidemic countries. In fact, cases of NOVC bacteraemia have been reported around the world, particularly in Asia, where cholera disease is widespread.^1^ Furthermore, NOVC infections are believed to be underdetected and underreported compared to *V. cholerae* O1/O139.^5^

**Fig. 2:**
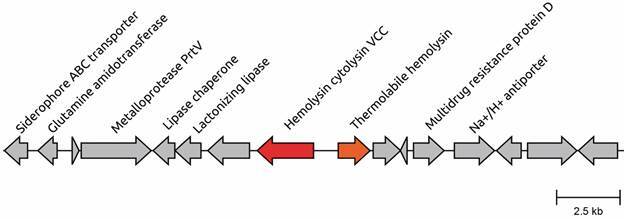
genomic context of *hly*A and *tlh* genes. Arrows represent predicted genes, where red and orange arrows indicate *hly*A and *tlh* genes, respectively.

Although the NOVC genomes lacked the toxigenic factors CTX and TCP, they shared several other virulence genes with epidemic *V. cholerae*, and even with *V. paracholerae* [Fig. 1 and Supplementary data (Table I)]. Some of these genes have been identified as predominant accessory virulence factors in NOVC and toxigenic strains, such as hemagglutinin protease (HapA), α-haemolysin (HlyA), repeats-in-toxin (RTX), toxin-regulating gene product (ToxR) and the type III and type VI secretion systems (T3SS and T6SS, respectively).^1^ Indeed, these virulence factors were prevalent in the phylogeny (Fig. 1), however, the T3SS was identified in 33% of NOVC genomes (8/24). Although the T3SS plays a role in the pathogenicity of *Vibrio parahaemolyticus* ^11^ and may enhance the virulence of NOVC strains, its limited distribution in NOVC genomes suggests that it is not a key factor in bacteraemia. Furthermore, we identified the thermolabile haemolysin (*tlh*) [Fig. 1 and Supplementary data (Table I)], another virulence factor also found in *V. parahaemolyticus*, which may play a fundamental role in infections by this species.^11^ Curiously, the *tlh* gene was located in tandem with the *hly*A gene (Fig. 2), another haemolysin, and is also shared with *V. paracholerae* and *V. cholerae* O1 genomes (Fig. 1).

The resistome of the NOVC genomes showed few genes [Supplementary data (Table II)]. Furthermore, none of them would be associated with resistance to the antibiotics typically used to treat Vibrio bacteraemia, which involves third-generation cephalosporins combined with tetracycline or doxycycline.^6^

Although NOVCs are responsible for the majority of cases of *V. cholerae* bacteraemia, it was not possible to clearly identify a virulence determinant that characterises this group. Future studies of these organisms based on other omics, such as transcriptomics, could provide new clues about the factors that modulate blood invasion. Moreover, it has been suggested that *V. paracholerae* can impact human health, at least causing bacteraemia. Furthermore, the presence of multiple lineages that cause bacteraemia suggests the lack of a specialised lineage. In fact, NOVC bacteraemia is strongly related to the consumption of contaminated water or food, in addition to some clinical presentations, particularly liver cirrhosis.^2^ ^,^ ^3^ Therefore, healthcare systems around the world need to be aware of this rare but growing problem.
